# Dynamic Methods for Childhood Hypoglycemia Phenotyping: A Narrative Review

**DOI:** 10.3389/fendo.2022.858832

**Published:** 2022-06-17

**Authors:** Alessandro Rossi, Martijn G. S. Rutten, Theo H. van Dijk, Barbara M. Bakker, Dirk-Jan Reijngoud, Maaike H. Oosterveer, Terry G. J. Derks

**Affiliations:** ^1^ Section of Metabolic Diseases, Beatrix Children’s Hospital, University of Groningen, University Medical Center Groningen, Groningen, Netherlands; ^2^ Department of Translational Medicine, Section of Pediatrics, University of Naples “Federico II”, Naples, Italy; ^3^ Laboratory of Pediatrics, University of Groningen, University Medical Center Groningen, Groningen, Netherlands; ^4^ Department of Laboratory Medicine, University of Groningen, University Medical Center Groningen, Groningen, Netherlands

**Keywords:** hypoglycemia, children, hepatic glycogen storage diseases, functional tests, fasting challenge, continuous glucose monitoring, stable isotopes

## Abstract

Hypoglycemia results from an imbalance between glucose entering the blood compartment and glucose demand, caused by a defect in the mechanisms regulating postprandial glucose homeostasis. Hypoglycemia represents one of the most common metabolic emergencies in childhood, potentially leading to serious neurologic sequelae, including death. Therefore, appropriate investigation of its specific etiology is paramount to provide adequate diagnosis, specific therapy and prevent its recurrence. In the absence of critical samples for biochemical studies, etiological assessment of children with hypoglycemia may include dynamic methods, such as *in vivo* functional tests, and continuous glucose monitoring. By providing detailed information on actual glucose fluxes *in vivo*, proof-of-concept studies have illustrated the potential (clinical) application of dynamic stable isotope techniques to define biochemical and clinical phenotypes of inherited metabolic diseases associated with hypoglycemia. According to the textbooks, individuals with glycogen storage disease type I (GSD I) display the most severe hypoglycemia/fasting intolerance. In this review, three dynamic methods are discussed which may be considered during both diagnostic work-up and monitoring of children with hypoglycemia: 1) functional *in vivo* tests; 2) *in vivo* metabolic profiling by continuous glucose monitoring (CGM); 3) stable isotope techniques. Future applications and benefits of dynamic methods in children with hypoglycemia are also discussed.

## Introduction

Appropriate investigation of the etiology and simultaneous management in children with hypoglycemia is paramount to prevent (irreversible) brain injury or even death (1), although controversy remains on the definition (e.g., diagnostic plasma glucose threshold, definition of at–risk neonates) of childhood hypoglycemia (2–4). From a pathophysiological perspective, hypoglycemia is caused by an imbalance between the amount of glucose molecules entering the blood compartment and the glucose clearance. A continuous supply of glucose is crucial for the function and development of the brain, because of the absence of sufficient cerebral energy stores in the form of glycogen, protein or fat. Glucose oxidation is responsible for ~ 70% of the energy production in the brain, whereas lactate and ketone bodies can function as important alternative fuels. Hypoglycemia causes energy deficiency in brain cells ultimately leading to brain cell death due to secondary molecular mechanisms, such as activation of neuronal glutamate receptors, oxidative stress, neuronal zinc release and activation of poly-ADP-ribose polymerase-1 (5, 6). Clinically, untreated hypoglycemia can lead to serious neurologic morbidity (encephalopathy, convulsions, coma, developmental delay), and even mortality (7). In individuals with a possible, underlying disorder, unrecognized hypoglycemia may accelerate and aggravate the metabolic decompensation resulting in poor outcomes.

Multiple metabolic pathways cooperate to maintain normal blood glucose concentrations in the fed and fasted state, such as glycogenolysis, gluconeogenesis, mitochondrial fatty acid oxidation, ketogenesis and ketolysis, ensuring energy homeostasis. These pathways are tightly regulated by hormonal (e.g., insulin, glucagon, cortisol, and growth hormone) and autonomic (e.g., catecholamines) responses (8) to maintain normoglycemia. Besides acquired conditions (e.g., insufficient supply/increased loss of nutrients, dysmaturity, drugs, hypothermia, sepsis), hypoglycemia may be explained by a genetic defect of these mechanisms that normally guarantee glucose homeostasis during feeding and fasting. Hypoglycemia is an important feature of several inherited metabolic disorders (IMDs) characterized by a history of childhood fasting intolerance (9).

Compared to adults, children are at an increased risk of developing hypoglycemia, as they have smaller hepatic glycogen and muscle protein stores, together with an increased energy expenditure and higher brain/body weight ratio (10). Consequently, most young children are not able to maintain normoglycemia during more than 24 hours of fasting (11–16). Studies with stable isotope techniques have quantified endogenous glucose production (EGP) in newborns (17) and older children (18). Huidekoper et al. designed a non-linear regression model for EGP from infancy to adulthood (19). **Table 1** illustrates that glucose homeostasis is tightly controlled in an age-dependent manner, with a small glucose pool in the blood compartment (row D) and high glucose turnover rate (row E).

**Table 1:** Relationship between age, blood glucose pool and endogenous glucose production (EGP).

A	Age (years)		1	3	5	10	15
B	Body weight (kg)^a^		13	18	23	35	48
C	Blood volume (ml)^b^		1,000	1,400	1,800	2,800	3,800
D	Blood pool of glucose (mg)		721	1,009	1,297	2,018	2,738
E	EGP	*(mg/kg/min)^c^*	8.2^d^	6.1	5.1	3.5	2.7
		*number of sugar cubes per day^d^*	34	35	37	40	41

a , based on ‘the APLS formula’ BW = [Age(years) + 4] x 2.5;

b , based on ‘the APLS formula’ BV = 80 ml/kg x BW(kg);

c , based on (19);

d , based on (20).

The most used diagnostic approach to childhood hypoglycemia consists of pattern recognition by combining a variety of information, including medical history (e.g., age at onset, relation with food), physical examination (e.g., growth charts, liver size), and laboratory tests. If critical samples (i.e. samples collected during metabolic stress) of plasma and urine can be obtained, static or single-point concentrations of biomarkers (e.g., blood glucose, lactate, gases, free fatty acids, ketones, acylcarnitines, amino acids, insulin, cortisol, growth hormone, and urine organic acids) can provide crucial information to establish an etiological diagnosis (21). When critical samples are not available or inconclusive, next-generation sequencing may further direct the diagnostic process. Several diagnostic algorithms exist for the clinical problem of childhood hypoglycemia (1, 3), but these schemes are often not sufficiently specific. Moreover, they underestimate the complexity of the swiftly changing clinical signs and concentrations of different fuels in the blood compartment. Hence, many hypoglycemic children do not fit these textbook schemes, and additional investigation is often required. In the end, an important group of individuals is diagnosed with idiopathic ketotic hypoglycemia (22, 23).

In this narrative review, we illustrate several dynamic methods which may be considered in the diagnostic work-up and monitoring of children with hypoglycemia. Three methods are presented: 1) functional *in vivo* tests; 2) *in vivo* metabolic profiling by continuous glucose monitoring (CGM); 3) stable isotope tracing, focusing on glycogen storage disease type I (GSD I). Future applications and benefits of dynamic methods in children with hypoglycemia are also discussed.

## Functional *in Vivo* Tests

For decades, functional *in vivo* tests have been used for the etiological clarification of fasting intolerance, unexplained hypoglycemia, and to diagnose specific IMDs (**Table 2**). In principle, these tests were particularly relevant as biochemical abnormalities might only appear intermittently in many IMDs. In times when diagnostic enzymatic studies and DNA studies were not routinely available, assessment of blood lactate curves after hexose (e.g. glucose, fructose and galactose) loading was used as a screening procedure in patients suspected of hepatic GSDs (24–26). The results of these tests combined with the assessment of response of blood glucose to glucagon injection provided major clues to identify the hepatic GSD subtype. As an example, the combination of increased blood lactate levels observed after oral glucose administration together with no response of blood glucose to intramuscular glucagon injection pointed to GSD III. Subsequently, the abnormally increasing lactate curves after ingestion of both fructose and galactose in GSD I patients has become one of the pillars, on which the current dietary recommendations are based. If performed, functional *in vivo* tests are conducted strictly adhering to a defined protocol to maximize the reliability and minimize the risks for patients. Functional *in vivo* tests should only be performed in experienced centers and under close medical supervision. Protocols for several functional *in vivo* (provocation and/or loading) tests can be found elsewhere (9, 27).

**Table 2:** Main *in vivo* functional tests used in children with hypoglycemia. Historical indications are shown.

Functional *in vivo* test	Historical indications
Explorative controlled fasting test	Assessment of fasting tolerance in situations of childhood hypoglycemia or fasting intolerance, before performing an extended controlled fasting test
Extended controlled fasting test	Clarification of hypoglycemia in FAOD, disorders of ketogenesis/ketolysis and some endocrinopathies
Intravenous glucagon test	Differentiation of hypoglycemia
Oral glucose loading test	Hypoglycemia or moderate/intermittent hyperlactatemia of unknown origin (e.g., hepatic GSDs, disorders of gluconeogenesis, PDH deficiency, hyperinsulinemia, mitochondrial disorders, SGLT1 deficiency)
Oral galactose loading test	SGLT1 deficiency Hepatic GSDs (GSD I excepted)
Oral fructose loading test	Hereditary fructose intolerance SGLT1 deficiency Differentiation of the different forms of PDH deficiency
Intravenous fructose loading test	Hereditary fructose intolerance Fructose-1,6-bisphosphatase deficiency
Oral protein/leucine tolerance test	Hyperinsulinism
Fat loading test	FAOD
Phenylpropionate loading test	Medium-chain acyl-coenzyme A dehydrogenase deficiency

FAOD, fatty acid oxidation disorders; GSDs, glycogen storage diseases; PDH, pyruvate dehydrogenase complex.

In theory, evaluation of metabolic changes in response to an explorative or extended controlled fasting test may be helpful to reach the etiological diagnosis in children with hypoglycemia. Fasting induces several hormonal and metabolic responses to ensure an endogenous supply of energy after cessation of exogenous intake (28). Due to the complexity of the (patho)physiological response to fasting, complete hormonal and metabolic investigations should be performed before and at the end of the monitored fasting. Spare serum/urine samples should also be stored for any additional tests to be performed afterwards. Bedside monitoring of these laboratory parameters, and in particular during the extended controlled fasting test, vital parameter monitoring and CGM should also be performed. Under these circumstances, the maximal duration of the fasting is based on the clinical history (expected fasting tolerance) and on the child’s age (usually 12-16 hours at 6-12 months, 18 hours at 1-2 years, 20 hours at 2-7 years, 24 hours in children > 7 years). The fasting should be stopped at any time if the glucose concentration is below 2.6 mmol/L or the patient develops symptoms of hypoglycemia (21).

Even before the era of expanded newborn screening and next generation sequencing, the diagnostic yield of fasting studies has been limited. Morris et al. confirmed an endocrine disorder (n=15) or an IMD (n=15) in a group of 138 patients undergoing fasting studies (29). Bappal and Mula-Abed diagnosed an endocrine disorder (n=21) or an IMDs (n=31) in 96 patients subjected to diagnostic fasting tests (30). Both studies did not provide detailed information on confirmatory diagnostic testing in their patients. More recently Graves et al. showed that 4/91 fasting challenges performed in children with suspected or documented hypoglycemia resulted in an etiological diagnosis (i.e., hepatic GSD, partial ACTH deficiency, panhypopituitarism, isolated central hypothyroidism) (31).

Although *in vivo* functional tests have the advantage of triggering metabolic stress in a supervised setting, they are burdensome and inconvenient for young patients and their parents. If IMDs are not yet excluded, these tests can cause serious complications in unexperienced hands (e.g., worsening of hypoglycemia, accumulation of toxic metabolites). Therefore, psychological burden due to unexpected hypoglycemia for patients and their families should be balanced against the stress and risks associated with *in vivo* functional testing. Additionally, *in vivo* functional tests pose a significant organizational and logistical burden (e.g., need for a fully equipped laboratory and a dedicated medical team) by including several samples to be collected at specific time points (before and after the challenge), under strictly supervised conditions, and requiring hospital admission.

Based on these considerations and because indications have become more restricted, most of the *in vivo* functional tests have fallen into disuse. A few tests are nowadays even abandoned and considered obsolete (9). Historical indications for functional *in vivo* tests have been shifted with the increased awareness for rare diseases, the development of diagnostic algorithms and refined basal metabolic investigations (such as organic acid analysis, plasma acylcarnitines, specific enzyme analysis, *in vitro* metabolic flux analysis in cultured skin fibroblasts), introduction of expanded population newborn screening, and implementation of next-generation sequencing. Still, some functional *in vivo* tests -with slight modifications - may be useful to provide phenotypic information to support genetic variant classification, or to define outcome in clinical trials. In such situations, these tests remain the major (monitoring) methods allowing the assessment of metabolic changes *in vivo* in response to external factors (e.g., metabolic stress, treatment). In addition, controlled fasting test may guide the diagnostic process in suspected congenital hyperinsulinism when critical samples are not available (e.g., molecular genetic testing, ^18^F-DOPA positron emission tomography) (32, 33).

## *In Vivo* Metabolic Profiling by Continuous Glucose Monitoring

Continuous glucose monitoring (CGM) constitutes another dynamic method in the diagnostic work-up and monitoring of children with hypoglycemia. Progress has been made since CGM was firstly described in 1979 for a patient with insulinoma (34). Modern CGM devices consist of a subcutaneous sensor and a receiver. A wearable sensor is attached to the skin and measures glucose (with variable frequency) through a subcutaneous needle inserted in the interstitial fluid. *Via* an enzymatic technology (glucose oxidase), the sensor generates an electric current proportional to the glucose concentration in the interstitium. The electric current flows through a continuously shifting algorithm to generate a glucose value which is eventually transmitted to the receiver (receiving unit or compatible smartphone). The algorithm is finetuned *via* calibration by patient or factory to ensure accuracy over the recording periods (35). Major advantages of CGM are the availability of real-time data, continuous data (density), alarming function, at home use, and data-sharing with caregivers and health care providers, and it being a relatively non-invasive technique (no or fewer finger sticks).

Currently, CGM devices are only licensed for patients with diabetes mellitus (DM), for whom CGM-related outcome parameters (e.g., time-in-range and time-above-range) are defined to guide dietary changes and/or medication adjustments (36). Additionally, CGM has been used in clinical research to assess glucose metabolism in various conditions, including acute coronary syndrome (37) and individuals following an intermittent fasting dietary regimen (38). Evidence on its benefit is also accumulating in endocrine disorders (39, 40) and IMDs associated with hypoglycemia (41), particularly in hepatic GSDs (**Table 3**).

**Table 3:** Previous studies using continuous glucose monitoring (CGM) in hepatic GSD patients.

Reference	Device	Country	Population (n. of patients)	Age (range in years)	GSD subtype
Hershkovitz ea. J Inherit Metab Dis. 2001 (42).	MiniMed (Medtronic)	Israel	4	2-15	Ia
Maran ea. Diabetes Metab Res Rev. 2004 (43).	Glucoday^®^ (Menarini)	Italy	4	14-47	Ia
			1	22	Ib
			1	10	III
White ea. J Inherit Metab Dis. 2011 (44).	iPro™ (Medtronic)	UK	1	6	0
			6	0-13	Ia
			2	0-3	Ib
			7	4-20	III
			4	5-16	IX
			2	2-24	XI
Kasapkara ea. Eur J Clin Nutr. 2014 (45).	MiniMed (Medtronic)	Turkey	15	2-18	Ia
			1		Ib
Herbert ea. J Inherit Metab Dis. 2018 (46).	Dexcom G4 Platinum (Dexcom)	USA	7	2-56	Ia
			2	9-17	Ib
			6	6-44	III
			5	7-17	IX
Kaiser ea. Mol Gen Metab. 2019 (47).	iPRO2^®^ (Medtronic) Guardian^®^ (Medtronic) FreeStyle Libre^®^ (Abbott)	Switzerland	12 2	11-49	Ia Ib
Peeks ea. J Inherit Metab Dis. 2021 (48).	Dexcom G6 (Dexcom) Dexcom G4 (Dexcom) Dexcom G6 (Dexcom)	Netherlands	1	9	Ia
			12	2-22	Ia, III, IX
			3	2-11	Ib

A significant correlation between CGM values and capillary glucose values has been found in patients with GSD I (42, 46). CGM can unveil both unrecognized (nocturnal) hypoglycemia and hyperglycemia and hence, provide information on the effect of the dietary regimen on glucose homeostasis in patients with hepatic GSDs (43). By providing visual glucose trends, CGM also has the advantage of generating dynamic, daily data compared to traditional single-point data. Thus, it can improve the home site monitoring (constituting an easier tool for patients compared to traditional methods) (44, 47) and the day-to-day healthcare for patients with hepatic GSDs (45, 46). CGM has also proven a reliable tool to assess the effect of novel/optimized dietary and/or medical treatments in patients with hepatic GSDs (48, 49). Additionally, it can assist the physicians in the assessment of children with hypoglycemia in combination with relevant information on personal and dietary history and appropriate biochemical investigations.

For patients with ketotic fasting intolerance (e.g., ketotic GSDs, idiopathic ketotic hypoglycemia, or any situation in which euglycemic ketonemia may occur, such as patients on ketogenic diets), parallel documentation of point-of-care capillary beta-hydroxybutyrate (BHB) concentrations by a portable ketone meter may provide important additional information. To the best of our knowledge, reference values for capillary BHB during the day are not available in the pediatric population, but information on BHB normal values after overnight fasting can be extrapolated from historical publications (**Table 4**). Most studies provide BHB concentrations after prolonged fasting, but recently, Parmar et al. demonstrated that serum point-of-care (POCT) BHB measurement using a precision Xtra meter are comparable to serum BHB concentrations in healthy children after an overnight fast (51). In 94 children (age range 6 months to 18.7 years) scheduled to undergo elective surgery, POCT BHB concentrations ranged from 0 to 1.1 mmol/l, but unfortunately, detailed information on the correlation between age and BHB concentrations was not provided in their study.

**Table 4:** Previous studies assessing ketones and glucose concentrations after prolonged fasting in healthy children.

Reference	Study population (n)	Age (years)	Duration of fasting (hours)	Descriptive statistics	Glucose concentrations (mmol/L)	Ketone body concentrations^1^ (mmol/L)	
Chaussain ea. J Pediatr. 1977 (11).	28	2 - 17	24	- Range	1.7 - 4.3 Blood sugar significantly correlated to age	Ketonuria ranging from 0 to +++ The extent of ketonuria seemed to decrease with age	
Wolfsdorf ea. Eur. J. Pediatr. 1982 (50)	23	1.9 - 16.7	24	- Mean and SD- 95% C.I. for BHB at various ages	3.3 ± 0.7	BHB: 2.8 ± 1.3; ACA: 0.4 ± 0.1 95% C.I. BHB.: - 3 years: 2.5-5.5 - 6 years: 2.0-5.0 - 9 years: 1.0-4.0 -12 years: 0.3-3.5 Negative correlation between plasma ketone bodies and age.	
Haymond ea. Metabolism. 1982 (12)	15	6.1 ± 0.8	30	- Mean and SD	2.9 ± 0.2	BHB: 3.7 ± 0.4; ACA: 1.3 ± 0.1 Negative correlation between plasma ketone bodies and blood glucose concentrations.	
Lamers ea. Clin Chim Acta.1985a (13)	72	3 - 15	14 (overnight)	- Mean and SD - p2.5-p97.5	4.34 ± 0.343.72-5.05	BHB: 0.22 ± 0.23; ACA: 0.10 ± 0.05 BHB: 0.07-1.58; ACA: 0.06-0.30 Negative correlation between plasma ketone bodies and age.	
Lamers ea. Clin Chim Acta.1985b (14)	13	3 - 5	24	- Median	3.5	BHB: 2.07; ACA: 0.55	
	58	6-15	40	- Mean and SD - p2.5-p97.5	3.44 ± 0.442.64-4.41	BHB: - 6-11 years: 3.15 ± 1.38; p2.5: 1.36; p97.5: 8.90 - 12 years: p2.5: 1.01; p97.5: 6.58 - 15 years: p2.5: 0.49; p97.5: 3.18 ACA: - 6-11 years: 0.66 ± 0.25; p2.5: 0.33; p97.5: 1.52 - 12 years: p2.5:0.28; p97.5:1.27 - 15 years: p2.5: 0.18; p97.5: 1.81 Negative correlation between plasma ketone bodies and age.	
Bonnefont ea. Eur. J. Pediatr.1990 (15)	12	< 1	15	- p10- p90	3.9 - 5.3	BHB: 0.1 - 1.0	
			20		3.5 - 4.6	BHB: 0.5 - 2.3	
			24		2.7 - 4.5	BHB: 1.1 - 2.8	
	27	1 - 7	15		3.5 - 4.8	BHB: <0.1 - 0.9	
			20		2.8 - 4.3	BHB: 0.8 - 2.6	
			24		2.8 - 3.8	BHB: 1.7 - 3.2	
	9	7 - 15	15		4.4 - 4.9	BHB: <0.1 - 0.3	
			20		3.8 - 4.9	BHB: <0.1 - 0.8	
			24		3.0 - 4.3	BHB: 0.5 - 1.3	
van Veen ea. Pediatrics. 2011 (16)	49	0 - 2	15	- Median - p10-p90	4.13.1 - 4.8	BHB: 1.00 0.22-2.34	ACA: 0.42 0.10-0.87
			20		3.32.8 - 3.9	BHB: 2.23 0.91-3.31	ACA: 0.42 0.38-1.05
	79	2 - 7	15		4.63.8 - 5.3	BHB: 0.30 0.03-1.26	ACA: 0.16 <0.05-0.43
			20		4.03.0 - 4.8	BHB: 1.19 0.36-2.56	ACA: 0.46 0.13-0.91
			24		3.83.0 - 4.8	BHB: 2.01 0.81-3.54	ACA: 0.710.27-1.14
	39	7 - 18	15		4.94.1 - 5.3	BHB: 0.19 <0.02-1.27	ACA: 0.12 <0.05-0.46
			20		4.23.3 - 5.2	BHB: 0.62 0.09-2.18	ACA: 0.29 0.09-0.81
			24		4.13.5 - 4.9	BHB: 1.31 0.32-2.46	ACA: 0.500.22-0.96
Parmar ea. JIMD Rep. 2021 (51)	94	0.5-18.7	Overnight	Range	3.9-6.7	BHB: 0.0 - 1.2	

^1^Assessed in plasma or serum if not otherwise stated.

^2^For each age and fasting duration group median values (top line) and p10-p90 range (bottom line) for BHB (left subcolumn) and ACA (right subcolumn) are shown.

ACA, acetoacetate; BHB, beta-hydroxybutyrate; p2.5, 2.5^th^ percentile; p10, 10^th^ percentile; p90, 90^th^ percentile; p97.5, 97.5^th^ percentile; SD, standard deviation; 95% C.I., 95% confidence intervals.

Currently, only the BHB meters can be used for minimally invasive home monitoring to guide dietary management after a working diagnosis has been established. To our opinion, the combination of real-time CGM with alarm function and a portable ketone meter may be used in children undergoing parallel diagnostic and therapeutic metabolic profiling for hypoglycemic disorders [(52) and **Table 5**]. A hybrid protocol combining the exploration of fasting tolerance (by CGM) with so-called *in vivo* metabolic profiling (BHB and possibly additional biochemical parameters) may replace the historical approach in patients suspected with hepatic GSDs. Such approach will likely further decrease the use of diagnostic *in vivo* fasting tests. Indeed, the combination of information collected through different testing approaches will likely represent the future strategy to characterize childhood hypoglycemia, including both the diagnostic and monitoring phase. Additional factors (e.g., experience of the medical team, patients’ needs, available resources, insurance policy) may result in variations in local practice, even between specialized centers.

**Table 5:** Metabolic profiling in two children with hepatic GSDs.

Case	Sample (time)	CGM Glucose (mmol/l)	Lactate (mmol/l)	BHB (mmol/l)	ALT (U/l)	AST (U/l)	Cholesterol (mmol/l)	Triglycerides (mmol/l)
		Reference value: –	Reference value: < 2.2	Reference value: < 0.42	Reference value: < 35	Reference value: < 45	Reference value: < 5.2	Reference value: < 2.0
1	A (14:09h)	5.1	5.4	0.06	1,220	1,503	2.9	7.54
	B (16:25h)	4.8	3.7	0.08	–	–	2.8	5.85
2	C (14:19h)	5.6	4.2	0.17	217	236	6.8	9.10
	D (06:30h)	4.2	–	0.2	–	–	–	–
	E (08:43h)	4.0	1.4	0.4	337	578	6.6	9.05
	F (11:00h)	3.9	–	1.3	–	–	–	–
	G (14:00h)	3.3	1.3	1.8	–	–	6.7	3.95

Case 1 was referred by the general pediatrician and seen at the metabolic outpatient clinic at the age of eight months with failure to thrive and suspicion of hepatomegaly. Sample A was randomly obtained two hours after the last meal and sample B was obtained pre-prandially. Subsequently, he was admitted at the ward and dietary management was titrated, guided by continuous glucose monitoring (CGM) and point-of-care (POCT) beta-hydroxybutyrate (BHB) measurements. Homozygosity for the c.4529dupA, p.Tyr1510* AGL variant confirmed the diagnosis GSD IIIa. Case 2 was referred by the general pediatrician and seen at the metabolic outpatient clinic on a Friday afternoon at the age of 3.3 years with protruding abdomen, hepatomegaly, and abnormal transaminase values. The initial history did neither indicate severe metabolic decompensations, nor fasting intolerance. Sample C was randomly collected, and the boy went home with his parents, while a food diary, CGM and POCT BHB measurements were started at home over the weekend. When he returned on Monday, the food diary documented that the patient drank milk and ate meals at night times (02:10h; 03:02h) and early in the morning (05:50h). At home, his POCT glucose and BHB concentrations were 3.7 mmol/l and 1.8 mmol/l respectively, after a pause 7h47min at night. At the ward after breakfast (sample D), during real-time monitoring with an alarming CGM and glucose/BHB POCT measurements (samples E and F), sample G was obtained pre-prandially. Subsequently, dietary management was titrated and homozygosity for the c.2126T>C, p.Leu709Pro PYGL variant confirmed the diagnosis GSD VI.–, not available.

## Stable Isotope Tracing to Characterize Childhood Hypoglycemia

The flow of metabolites through a (disrupted) metabolic pathway is determined by both the rate of supply of substrates and the residual activities of the enzymes in the pathway. In patients with IMDs, changes in the distribution of fluxes occur secondary to enzyme defect(s) and/or altered substrate availability. By assessing the flow of metabolites through specific metabolic pathways *in vivo*, fluxomics can provide an overall description of tissue phenotypes in a relatively simple manner (53). (Radioactive or stable) isotope tracers have been used since 1930s to characterize such changes in cellular and whole-body metabolism (54). Stable isotopes techniques are well established methods in fundamental/translational research to assess metabolic fluxes/changes (55, 56). Proof-of-concept research has also shown the potential clinical (research and care) application of stable isotopes to characterize the biochemical and clinical phenotype of IMDs associated with hypoglycemia (57, 58). An overview on current evidence paving the path for future use of stable isotope techniques in diagnosis and monitoring of IMDs characterized by hypoglycemia is provided, with specific focus on GSD I.

### Principles of Stable Isotopes Methods

The core assumption of isotopic methods is that a tracer [i.e., a substance that can be administered in order to follow a natural substance (tracee)] is metabolically indistinguishable from the tracee (59). Ideally, the amount of the administered tracer is sufficiently low to avoid any effect on the physiological process investigated (59). Flux calculations are often performed when the system is in a steady state (i.e., a condition in which the tracer is infused continuously and its dilution in the analyzed compartment (e.g., plasma) remains constant over time). In this condition, blood samples are collected before the tracer intravenous infusion (after a priming dose) and several times after achieving the steady state (60).

Two types of tracers can be used: those that contain radioisotopes or those that contain stable isotopes. Radioisotopes are isotopes which release radiation as a by-product of their decay. The energy released during the decay can be measured, following the path of the tracer from reactants to products. Although such tracers have been widely used up to the 1970s (61), the health risk associated with radioactive tracers in humans and the development of affordable mass spectrometers led to the greater use of stable isotope tracers (62). Stable isotopes do not emit radiation and can be easily detected by mass spectrometry. Stable isotope tracers employed to study carbohydrate metabolism usually contain a hydrogen isotope (e.g., D-[6,6-^2^H_2_]-glucose, or D-[1-^2^H]- galactose) or a carbon isotope (e.g., uniformly labelled glucose D-[U-^13^C]-glucose or [2-^13^C]- glycerol). Since tracer loss without net conversion to product, that may be due to recycling (for example in case of recycling of D-[2-^2^H_2_]-glucose by conversion of glucose-6-phosphate to fructose-6-phosphate and back), may occur at specific metabolic steps, appropriate selection of the tracer is crucial for accurate analysis (63). The use of D-[6,6-^2^H_2_]-glucose or D-[U-^13^C]-glucose may in part overcome the pitfalls associated with other tracers (64). Given that each stable isotope is naturally found in the environment and in the body at a low concentration, it is important to take into account its natural abundance in order to determine the degree of tracer enrichment (65). Also, it is necessary that the degree of enrichment is high enough to be measured. The enrichment is calculated as a fraction: [tracer]/([tracer]+[tracee]) and is usually expressed as mole percent excess (MPE) (63).

For glucose metabolism during fasting, at biological steady state the rate of appearance of glucose into the circulation (R_a_) represents endogenous glucose production (EGP) in the absence of an additional input of glucose. Moreover, at this steady state R_a_ equals the rate of glucose disposal (R_d_). Experimentally, R_a_ is estimated during a continuous infusion of a solution of labeled glucose from the measured dilution of stable isotope labeled glucose (for example D-[U-^13^C]-glucose) in the pool of plasma glucose at isotopic steady state. In general, the rate of infusion of labeled glucose (I) cannot be neglected in comparison with R_a_. Therefore, during a stable isotope tracer infusion experiment the total rate of appearance of glucose (R_t_) into the circulation is considered as, the sum of R_a_ and I: R_t_ = R_a_ + I = R_d_. At isotopic steady state, the R_t_ can be calculated from the dilution of infused labelled glucose in the plasma glucose pool, as the ratio of fractional isotopologue enrichment of infusate over the fractional isotopologue enrichment of blood glucose, multiplied with the infusion rate. When D-[U-^13^C]-glucose, with 6 ^13^C carbon atoms, is applied as a glucose label ${\text{R}}_{\text{t}}=\left({\text{M}}_{\text{infusate}}^{+6}/{\text{M}}_{\text{blood glucose}}^{+6}\right)*\text{I}$ . Then R_a_ can be calculated as: ${\text{R}}_{\text{a}}{\text{= R}}_{\text{t}}-\text{I = }\left(\left({\text{M}}_{\text{infusate}}^{+6}/{\text{M}}_{\text{blood glucose}}^{+6}\right)-1\right)*\text{I}$ . (59).

However, there are situations in which additional variables play a role and in which tracer enrichment can vary (non-steady state). For example, inflow of glucose (e.g., from the intestine in the post-absorptive state, or additional glucose infusion) or hormonal response (e.g., insulin, cortisol) drives glucose through different compartments before entering (and possibly re-entering) the bloodstream. Such complex conditions can still be simplified to a single-compartment model by including a pool volume of the total blood glucose pool and the changes in the [tracer]/([tracer]+[tracee]) enrichments over time in the calculations (66). Still, changes in the pool volume over time and equilibration rate of the tracer between compartments can result in aberrant results when using this approach. Frequent sampling and appropriate data fitting can minimize such errors (59). Alternatively, the pool volume can be measured (e.g., by infusing two or even three different isotopes), requiring more complex experimental designs (67).

### Stable Isotopes Techniques to Study Conditions Associated With Hypoglycemia

Stable isotopes have been widely used to study the *in vivo* dynamics of glucose metabolism and conditions associated with hypoglycemia. As previously mentioned, R_a_ and R_d_ are the key variables when performing tracer studies. For blood glucose, R_a_ reflects the EGP in fasted conditions in the absence of any exogenous input [e.g. (e.g., glucose influx from the intestine, or additional unlabeled glucose infusion]. Physiologically, two main biochemical processes, namely gluconeogenesis and glycogenolysis, account for EGP (68). Conversely, R_d_ represents glucose uptake from the blood by the liver and other organs, and is determined by several metabolic pathways, including glycolysis and glycogen synthesis.

In 1977, Bier et al. investigated glucose turnover by using stable isotope tracers for the first time (20). Particularly, D-[6,6-^2^H_2_]-glucose intravenous infusion allowed quantification of EGP in children (with either hypoglycemia or normal glucose homeostasis) and (normoglycemic) adults, revealing that 1) physiologically EGP is higher in children than adults when normalized for body weight and 2) a linear correlation between EGP and brain weight exists in children (20). This study set the stage for further in-depth research on childhood hypoglycemia assessing glucose kinetics with stable isotope methods (69–71). D-[6,6-^2^H_2_]-glucose intravenous infusion has been used to investigate glucose kinetics in idiopathic ketotic hypoglycemia. Particularly, it has been clarified that this condition is due to impaired EGP (rather than increased glucose utilization) (72) secondary to insufficient increase in gluconeogenesis (71). In a single patient with congenital hyperinsulinism increased EGP and glycogenolysis after glucagon administration were demonstrated. Specifically, increased glucose R_a_ (assessed by D-[6,6-^2^H_2_]-glucose intravenous infusion) and decreased glycerol R_a_ [assessed by [2-^13^C]-glycerol intravenous infusion (73)] were noted. Additionally, various techniques to estimate *in vivo* rates of gluconeogenesis and/or glycogenolysis with stable isotopes have been developed (74).

As the liver accounts for ∼80% of EGP (with the rest accounted for mainly by the kidneys), a particular interest has developed to assess glucose kinetics with stable isotopes tracers in IMDs in which hypoglycemia is mainly due to the enzyme defect in the liver. A detailed report on the use of stable isotope methodology in IMDs goes beyond the scope of the present work [see for a review (75)]. Specific results on selected IMDs associated with hypoglycemia are presented here, with a special focus on GSD I.

Flux analysis in a mouse model of medium chain acyl-CoA dehydrogenase deficiency (MCADD) has shown that pharmacological inhibition of mitochondrial fatty acid oxidation does not affect EGP (76). Insufficient increase of gluconeogenesis upon hypoglycemia has been observed in long-chain acyl-CoA dehydrogenase-deficient mice (77). Observations on a patient with propionic acidemia showed elevated carbohydrate utilization both at rest and during exercise, suggesting that these patients rely more on carbohydrates as energy source than healthy volunteers (78). Research on hereditary fructose intolerance (HFI) showed that aldolase B contributes for around 50% of the fructose to glucose conversion in healthy subjects and that children with HFI exhibit a significantly lower conversion of fructose to glucose (79).

Several studies have made use of stable isotope tracer methodology to investigate glucose kinetics in GSD I, both in rodent models (80, 81) and humans (57, 82–88). GSD I (MIM# 232200) is an inherited disorder of glycogen metabolism due to a defect in the glucose 6-phosphatase (G6Pase) system. Defects either in the catalytic subunit (G6Pase-α, *G6PC1*) or in the microsomal glucose 6-phosphate transporter (G6PT, *SLC37A4*) cause GSD Ia and GSD Ib, respectively. Impaired glycogenolysis and gluconeogenesis result in fasting intolerance with hypoglycemia, elevated lactate, metabolic acidosis and secondary metabolic derangements. Uncooked cornstarch (UCCS) or extended-release cornstarch (Glycosade^®^) and/or continuous gastric drip-feeding is the cornerstone of the treatment (52).

Assessing EGP in GSD I has been one of the key research objectives for decades (57, 80, 82–88). After evidence on residual EGP was first provided in two patients with GSD I (89), several studies have made use of stable isotope methods to quantify glucose production in GSD I patients (57, 82–88). Those studies showed that, despite G6Pase deficiency, a limited (up to ∼60% of normal) capacity of EGP could be found in GSD Ia. Yet, estimated EGP varied considerably among patients. In some of these studies EGP was likely overestimated due to the contribution of dietary glucose or intravenous/intragastric glucose infusions, which caused total glucose R_a_ to exceed EGP. The reason why there is still residual EGP in GSD I is still not clear. Various mechanisms have been proposed, including residual G6Pase activity (90), extra-hepatic/renal/intestinal EGP (91), increased debrancher enzyme (AGL) activity, and increased acid alpha glucosidase (GAA) activity (82). Studies on rodent models have contributed to further delineate the (patho)physiological basis of this process. Stable isotope tracer methodology to investigate glucose kinetics in animal models for GSD I was first applied in an acute model of GSD Ib in fasted rats using the pharmacological G6PT-inhibitor S4048. The method applied consisted of an infusion of stable isotopes D-[U-^13^C]-glucose, [2-^13^C]-glycerol, and D-[1-^2^H]-galactose, as well as paracetamol, to quantify hepatic glucose and glycogen metabolism (80). Later, this same methodology was applied to mice with hepatocyte-specific *G6pc* deficiency, a model for hepatic GSD Ia (81), which retained about 30% of EGP compared to their wild-type littermates. Primary hepatocytes from these animals showed that residual glucose production (~20% of wild-type littermates) was almost completely abolished upon treatment with an α-glucosidase inhibitor, which inhibits both AGL and GAA. This indicates that either increased glycogen debranching and/or lysosomal glycogen breakdown accounts for the production of free glucose by the GSD Ia liver and hence could explain the residual EGP in GSD I (81). Nonetheless, research assessing the utilization of (various types of) cornstarch in hepatic GSD patients (92, 93) supports the extensibility of stable isotopes methods also to human research.

## Discussion and Future Perspectives

Although the past years have witnessed major advances in understanding the pathophysiology of disorders associated with hypoglycemia, relevant limitations in assessment and monitoring strategies remain. The current diagnostic approach to children presenting with hypoglycemia includes, besides medical/dietary history and clinical examination, biochemical tests (performed on “critical samples”) and eventually genetic confirmation (21). However, critical samples or reliable biochemical data are not always available or may not provide conclusive information. Also, traditional biochemical markers are static parameters and do not always appear sufficient to explain the phenotypic variability in patients with IMDs associated with hypoglycemia (94). Furthermore, those biomarkers may not adequately reflect the dynamic pathophysiological processes taking place in response to external factors such as metabolic stressors or treatment. Thus, there remain a group of patients with unsolved diagnostic and/or monitoring questions, that may be addressed by dynamic methods, such as *in vivo* functional tests, CGM and stable isotope tracing.

Functional *in vivo* tests have the advantage of characterizing metabolic pathways, such as glycogenolysis, gluconeogenesis, mitochondrial fatty acid oxidation, ketogenesis, and ketolysis, in a dynamic fashion. Yet, their use is progressively decreasing. Therefore, we do not recommend performing such tests in unexperienced centers. Several functional *in vivo* tests (e.g., controlled fasting challenge) are currently designed to monitor management after novel, innovative treatments (NCT03517085, NCT03665636, NCT05095727, NCT NCT04538989, NCT04720859, NCT03761693) in centers of expertise. Fasting test may still play a role in the diagnostic process of congenital hyperinsulinism. Yet, the development of safer and simpler diagnostic and monitoring tools for clinical research/care remains a compelling need (95).

CGM constitutes a highly informative technique to (non-invasively) monitor glucose levels in a dynamic fashion in patients with hypoglycemia (39–41, 45). Although physicians working in centers of expertise have become experienced with the CGM technology, its use in less experienced centers remains limited. Therefore, knowledge dissemination on the CGM technology and data interpretation is paramount. In fact, given its relatively low organizational burden and invasiveness, CGM may constitute a valuable technique. Ideally, individual patients’ CGM results could be compared with (1) the patient’s historical CGM data before and after intervention, and CGM data from both (2) an age-matched disease cohort and (3) age-matched healthy controls. Shah et al. recently reported CGM profiles from 153 nonpregnant, healthy, nondiabetic children and adults (age ≥6 years) with nonobese body mass index, using a blinded Dexcom G6 CGM, with once-daily calibration, for up to 10 days (96). Advanced CGM data analysis represents a promising strategy for minimally invasive patient monitoring (48). However, several challenges constrain CGM use in patients with hypoglycemia. First, neither general agreement on relevant CGM-related outcome parameters nor reference values for such parameters exist for patients with IMDs. Ideally, individual patients’ CGM results would be comparable once these issues have been solved. Second, the lag time (i.e., the time delay between a change in plasma glucose and the reporting of this change by the CGM device) may affect CGM reliability in patients experiencing sudden drops in glucose concentrations, as in hepatic GSDs. Third, most CGM devices do not contain predictive alarms (i.e., predicting an upcoming low glucose event using trend information) and rely upon the user to identify hypoglycemia and make manual adjustments. Also, current predictive alarms are based on a linear function of glucose trends, which may not adequately reflect the exponential decrease observed in hypoglycemic GSD patients (97). Likely the progress in machine learning and deep learning approaches and artificial neural networks will develop reliable algorithms for hypoglycemia prediction in patients with IMDs (35). Fourth, some devices require fingerprick calibration, which currently prioritizes hyperglycemia over hypoglycemia, thus constituting a potential additional source of inaccuracy, particularly a in the low range of blood glucose (35). Notably, most recent devices such as Dexcom G6 and Freestyle Libre are factory-calibrated (98). Fifth, as no specific CGM device is formally licensed for patients with IMDs, several types of CGM devices are used (**Table 3**) presenting with different functionalities, advantages, and limitations. Additionally, data management applications currently vary, depending on the type of CGM device installed and interconnectivity between CGM software packages, and digital health records are lacking. Harmonization of data management applications as well as improved interconnectivity between CGM software packages and data health records are strongly warranted to facilitate data sharing among healthcare professionals and beyond towards citizen science applications. Sixth, while a 14-day data collection is recommended in DM patients (99), the number of measurements required to generate reliable CGM profiles in patients with IMDs is still unknown.

Stable isotope techniques appear attractive as they give access to the *in vivo* dynamics of hypoglycemia from a pathophysiological perspective. In principle, this may prevent a late diagnosis in patients presenting with milder phenotypes (100, 101). Yet, this technique has been explored experimentally in a limited number of conditions (e.g., idiopathic ketotic hypoglycemia, GSD I, congenital hyperinsulinism), but it is currently not clinically implemented, neither applied in clinical trials. Potential clinical benefit of this approach includes the definition of an easy and safe method to guide the diagnosis of patients with hypoglycemia and to improve patient monitoring, possibly constituting an additional tool to assess the effect of novel treatments (e.g., gene therapy, mRNA therapy). Given the safety of this methodology, it appears well suited for clinical trials in humans. Traditionally, venous blood samples are collected before the intravenous tracer infusion (after a priming dose) and several times after achieving the steady state (60). Such a procedure may potentially hamper the translation of this approach into clinical research/care in its current form. Previous research has shown the reliability of dried blood spot (DBS) sampling (compared to traditional venous sampling) in mice by tail bleeding to assess glucose kinetics following infusion of D-[U-^13^C]-glucose, D-[1-^2^H]-galactose, and [2-^13^C]-glycerol (102) or after an intraperitoneal injection of D-[6,6-^2^H]-glucose (58). Although these findings pave the way for possible oral (non-steady-state) administration of stable isotope tracers to humans (who are metabolically in a steady state, i.e. upon fasting without any unknown (unmeasured) and/or non-steady additional exogenous glucose input), the effect of this administration route on the tracer bioavailability remains unknown. Future studies exploring this approach in humans are warranted. Additionally, the use of stable isotope tracers in animal models can guide further dissection of pathophysiological derangements occurring in IMDs.

In addition to functional *in vivo* tests, metabolic profiling by CGM and stable isotope techniques, the microdialysis technique, at least in theory, would enable continuous sampling of multiple metabolites from the extracellular space from various tissues, such as blood, adipose tissue, or brain in experimental models (103, 104). The combination of stable isotope tracing and microdialysis in animal models appears intriguing (105). As microdialysis allows not only glucose monitoring (106, 107) but also sampling of other metabolites (e.g., glycerol, acylcarnitines) (108), it has a great potential for experimental research in patients with IMDs. Still, there are currently no commercial systems available.

## Author Contributions

AR, MGSR, MHO and TGJD wrote the first version of the manuscript. THvD, BMB, D-JR and TGJD critically reviewed the manuscript. AR drafted and wrote the final version of manuscript. TGJD critically reviewed the final version of the manuscript. All authors substantially contributed to the work and were involved in (a) conception and design of the study and/or analysis and interpretation of data, and (b) revising the article critically for important intellectual content. All authors approved the final manuscript as submitted and agree to be accountable for all aspects of the work. All authors confirm the absence of previous similar or simultaneous publications. All authors contributed to the article and approved the submitted version.

## Conflict of Interest

The authors declare that the research was conducted in the absence of any commercial or financial relationships that could be construed as a potential conflict of interest.

The handling editor declared a past co-collaboration with the authors.

## Publisher’s Note

All claims expressed in this article are solely those of the authors and do not necessarily represent those of their affiliated organizations, or those of the publisher, the editors and the reviewers. Any product that may be evaluated in this article, or claim that may be made by its manufacturer, is not guaranteed or endorsed by the publisher.
